# Mitotic catastrophe and cell cycle arrest are alternative cell death pathways executed by bortezomib in rituximab resistant B-cell lymphoma cells

**DOI:** 10.18632/oncotarget.14405

**Published:** 2016-12-31

**Authors:** Juan J. Gu, Gregory P. Kaufman, Cory Mavis, Myron S Czuczman, Francisco J. Hernand ez-Ilizaliturri

**Affiliations:** ^1^ Department of Medicine, Roswell Park Cancer Institute, Buffalo, NY, USA; ^2^ Department of Immunology, Roswell Park Cancer Institute, Buffalo, NY, USA; ^3^ Department of Internal Medicine, Mayo Clinic, Rochester, MN, Celgene Corporation, Summit, NJ, USA

**Keywords:** bortezomib, B-cell lymphoma, rituximab

## Abstract

The ubiqutin-proteasome system (UPS) plays a role in rituximab-chemotherapy resistance and bortezomib (BTZ) possesses caspase-dependent (i.e. Bak stabilization) and a less characterized caspase–independent mechanism-of-action(s). Here, we define BTZ-induced caspase-independent cell death pathways. A panel of rituximab-sensitive (RSCL), rituximab-resistant cell lines (RRCL) and primary tumor cells derived from lymphoma patients (*N* = 13) were exposed to BTZ. Changes in cell viability, cell-cycle, senescence, and mitotic index were quantified. In resting conditions, RRCL exhibits a low-proliferation rate, accumulation of cells in S-phase and senescence. Exposure of RRCL to BTZ reduces cell senescence, induced G2-M phase cell-cycle arrest, and is associated with mitotic catastrophe. BTZ stabilized p21, CDC2, and cyclin B in RRCL and in primary tumor cells. Transient p21 knockdown alleviates BTZ-induced senescence inhibition, G2-M cell cycle blockade, and mitotic catastrophe. Our data suggest that BTZ can induce apoptosis or mitotic catastrophe and that p21 has a pivotal role in BTZ activity against RRCL.

## INTRODUCTION

The integration of rituximab to chemotherapy regimens has improved outcomes of diffuse large B-cell (DLBCL) and indolent B-cell lymphoma [1–4]. Acquirement of resistance in DLBCL is a major clinical problem [5]. Previously, we found that rituximab exposure leads to chemotherapy resistance suggesting the existence of shared pathways of resistance [6, 7]. Rituximab/chemotherapy resistant cells were found to have an upregulated ubiquitin-proteasome system (UPS) and deregulated apoptotic machinery [6, 8].

Bortezomib (BTZ) induced additive and/or synergistic anti-tumor effects to various chemotherapy agents or rituximab in preclinical studies [9]. These observations lead to the evaluation of BTZ in combination with rituximab and/or systemic chemotherapy in patients with previously untreated or relapsed/refractory indolent or aggressive B-cell lymphoma [10–13]. Because of the variable degree of success observed, further research evaluating BTZ mechanisms-of-action is needed in order to optimize its anti-tumor activity.

The UPS regulates numerous proteins involved in the cell cycle, apoptosis, cell proliferation and differentiation. BTZ has been shown to induce apoptosis, [14–16] to arrest cell proliferation,17,18 inhibit angiogenesis,19,20 and disrupt the cell-cycle in cancer cells [15, 21, 22]. BTZ's contribution to apoptosis in lymphoma depends on the transcription regulation and/or inhibition of the proteasome degradation of various anti- or pro-apoptotic proteins [8]. A major downstream target of BTZ is nuclear factor kappa-light-chain-enhancer of activated B-cells (NFκB). BTZ inhibits NF-κB activity through stabilization of the IκB kinase complex preventing the transcription of key regulatory anti-apoptotic genes and promoting programmed cell-death [23–25]. We demonstrated that BTZ inhibited the proteasome degradation of Bak and induced apoptosis in rituximab-chemotherapy pre-clinical models [7] Neither transient knockdown of Bak or caspase inhibition fully rescued rituximab-chemotherapy resistant cell lines (RRCL) to BTZ cytotoxic effects, suggesting the execution of alternative cell-death pathways [8].

Caspase independent pathway(s) triggered following UPS inhibition are poorly characterized. BTZ prevents the proteasome degradation of key cell-cycle regulatory proteins such as p21, the mouse double minute 2 homolog (MDM2), cyclin-B, cyclin-A, and the cyclin-dependent kinase-1 (cdk1 or CDC2)/cyclin-B complex. The accumulation of these regulatory proteins leads to cell-cycle arrest, apoptosis, or mitotic catastrophe in various cancer cell-lines [15, 26–31]. In the present contribution, we studied and characterized caspase-independent death pathways executed in BTZ-exposed RRCL. We demonstrate that besides altering the Bcl-2 family member cellular levels, BTZ induces G2/M cell cycle arrest followed by mitotic catastrophe in rituximab/chemotherapy resistant pre-clinical models. Our data suggests that p21 plays an important role in caspase-independent pathways triggered by BTZ exposure. In addition, we demonstrated that BTZ enhanced killing effect of mitotic-specific chemotherapy agents. Our findings provide valuable information into BTZ's molecular mechanism-of-action, and may aid in future clinical trial design for relapsed/refractory lymphoma.

## RESULTS

### Distinct patters of cell cycle, proliferation, and senescence in RRCL

**Figure 1:**
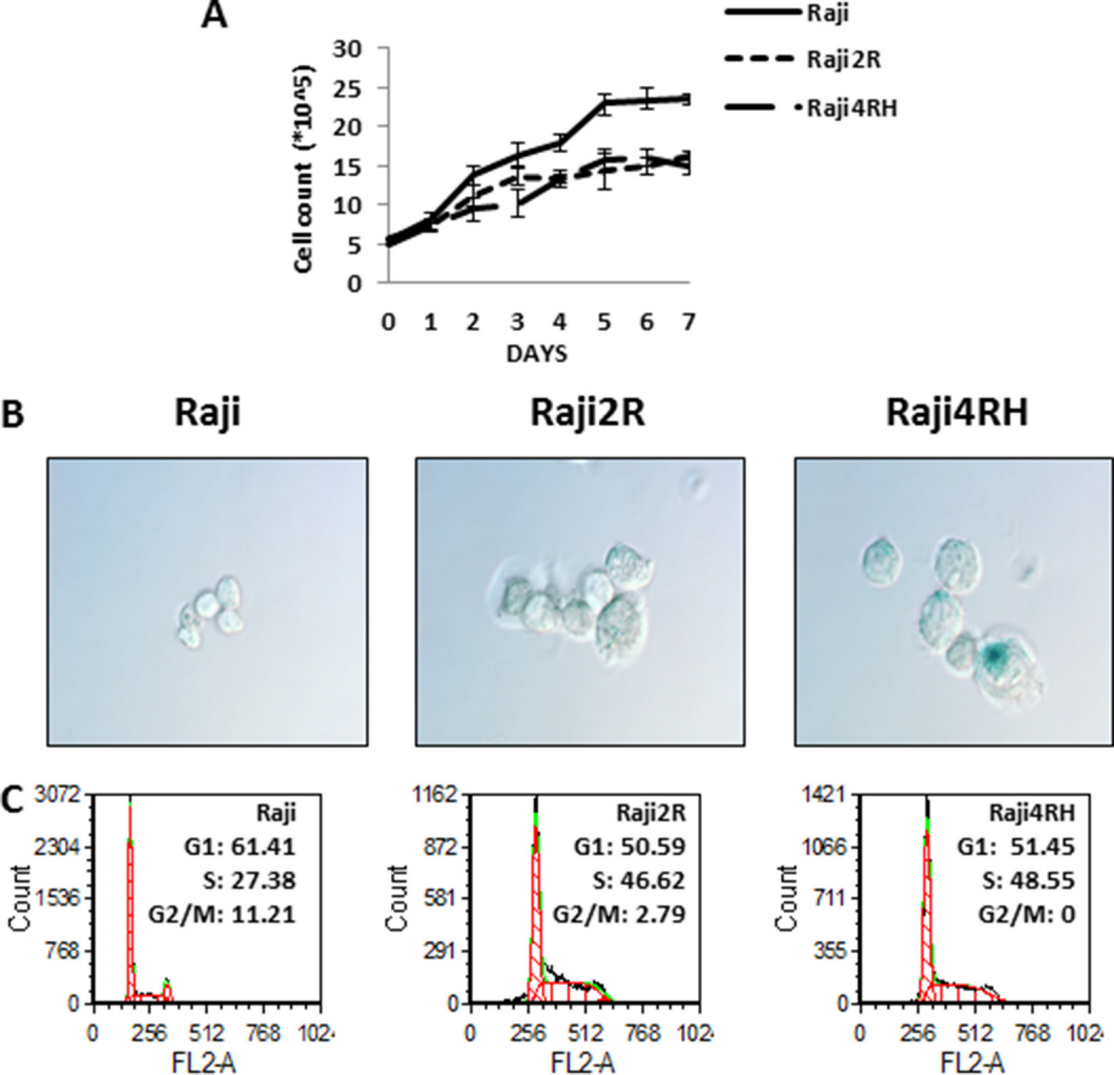
Differences in growth curves, senescence and cell cycle were found between RSCL and RRCL (**A**) Raji family cells were seeded at the same density (5 × 105/ml) and cell numbers were counted each day at the same time point up to one week. These experiments represented the mean of three independent experiments. Data shown are average of three independent experiments ± SD. (**B**) morphology and senescence cells (blue) of RSCL and RRCL. Senescent cells that exhibit higher amounts of B-galactosidase activity were detected by the Senescent Cells Staining Kit (Sigma) primarily in Raji cells. Original magnification, 60X. (**C**) cell cycle differences were determined by flow cytometry in the Raji, Raji2R and Raji4RH cells, respectively.

When compared to RSCL (Raji cells), Raji 2R and Raji 4RH cell lines (RRCL) are morphologically larger and proliferate at a slower pace under non-stressing culture conditions (Figure 1A). Differences in the β-galactosidase activity, a surrogate of cellular senescence, between RRCL and RSCL were also observed. RRCL exhibited higher β-galactosidase activity than RSCL (Figure 1B). RRCL (Raji 2R and Raji 4RH) were primarily distributed in the S-phase of the cell cycle in contrast to RSCL (Raji cells) (Figure 1C). In addition, we found that RRCL overexpressed Cyclin B1, CDK7, CDK2 and to a lesser degree Wee1 and down-regulated CDK6 when compared to RSCL. The changes in cell cycle regulatory proteins observed favored S-phase accumulation in RRCL (Figures 1 and 3A).

**Figure 2:**
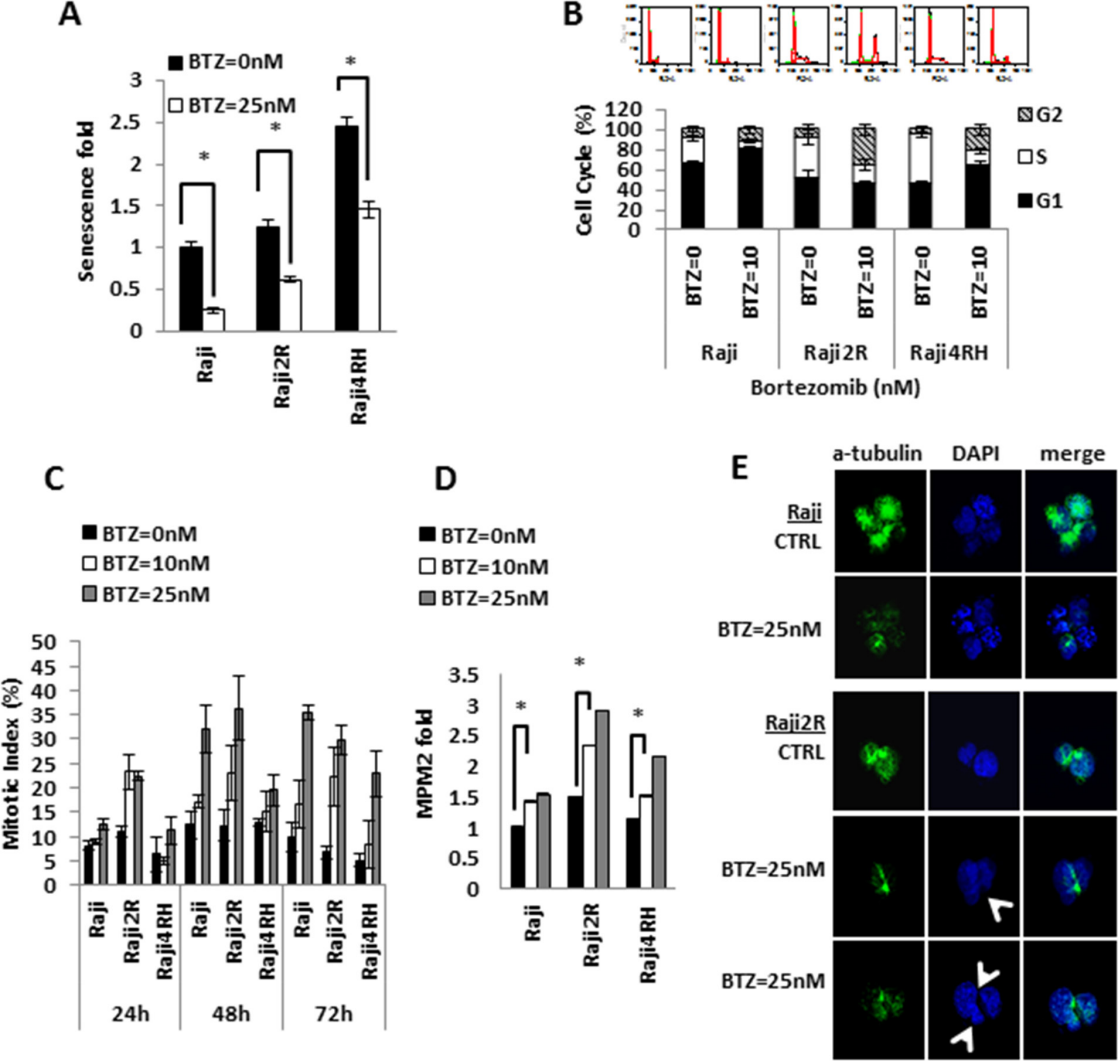
Bortezomib exposure results in variable degrees of senescence inhibition, G2/M arrest and mitotic catastrophe in RSCL and RRCL (**A**) Senescence fold of RSCL and RRCL was determined after cells exposed to BTZ 25 nM for 24 hrs. Fold of senescence were all normalized with Raji untreated control. Columns represent the means of three independent experiments; bars, SD. *P < 0.05. (**B**) Cell cycle profiles were evaluated after exposure to BTZ in RSCL and RRCL. Cells untreated or treated with 10 nM BTZ for 24 hrs were fixed, stained with PI and analyzed by flow cytometry as described in the Material and Methods. Upper, the experiment shown is a representative example from six different experiments. Lower, quantification of cell cycle experiments (n = 3) with Raji family cells treated with BTZ 10 nM for 24 hrs and graphed as mean +/– SD. (**C**) Mitotic index of cells treated with BTZ. For quantitative assessment of M-phase arrest by BTZ treatment, cells were exposed to indicate concentrations of BTZ for 24, 48, 72 hrs. After treatment, cells were harvested and stained with Wright-Giemsa dye solution. The percentage of mitotic cells were measured by counting stained cells/total cell numbers. Each bar represents of mean ± SD of three independent experiments. (**D**) MPM2 staining data revealed BTZ dose-dependent mitotic induction in RSCL and RRCL. Cells were treated with BTZ 10 nM, 25 nM for 24 hrs, and collected. After fixing with 2% formaldehyde and permeabilization, cells were incubated with anti-phospho-Ser/Thr-MPM2 overnight, and measured by flow cytometry analysis. Data were normalized to Raji untreated cells. The differences between BTZ untreated and treated cells are significant in RSCL and RRCLs, *P < 0.01. Each bar represents of mean ± SD of three independent of experiments. (**E**) BTZ induces mitotic catastrophe in RRCL, but not in RSCL. Mitotic spindles were visualized with an FITC-conjugated a-tubulin antibody (green) and nuclear was stained by DAPI (blue) under a fluorescence microscope. Control cells showed a single round nucleus or normal chromatid separation. Btz treated Raji2R cells demonstrated various abnormalities resulting in asymmetrical distribution of DNA (arrow).

**Figure 3:**
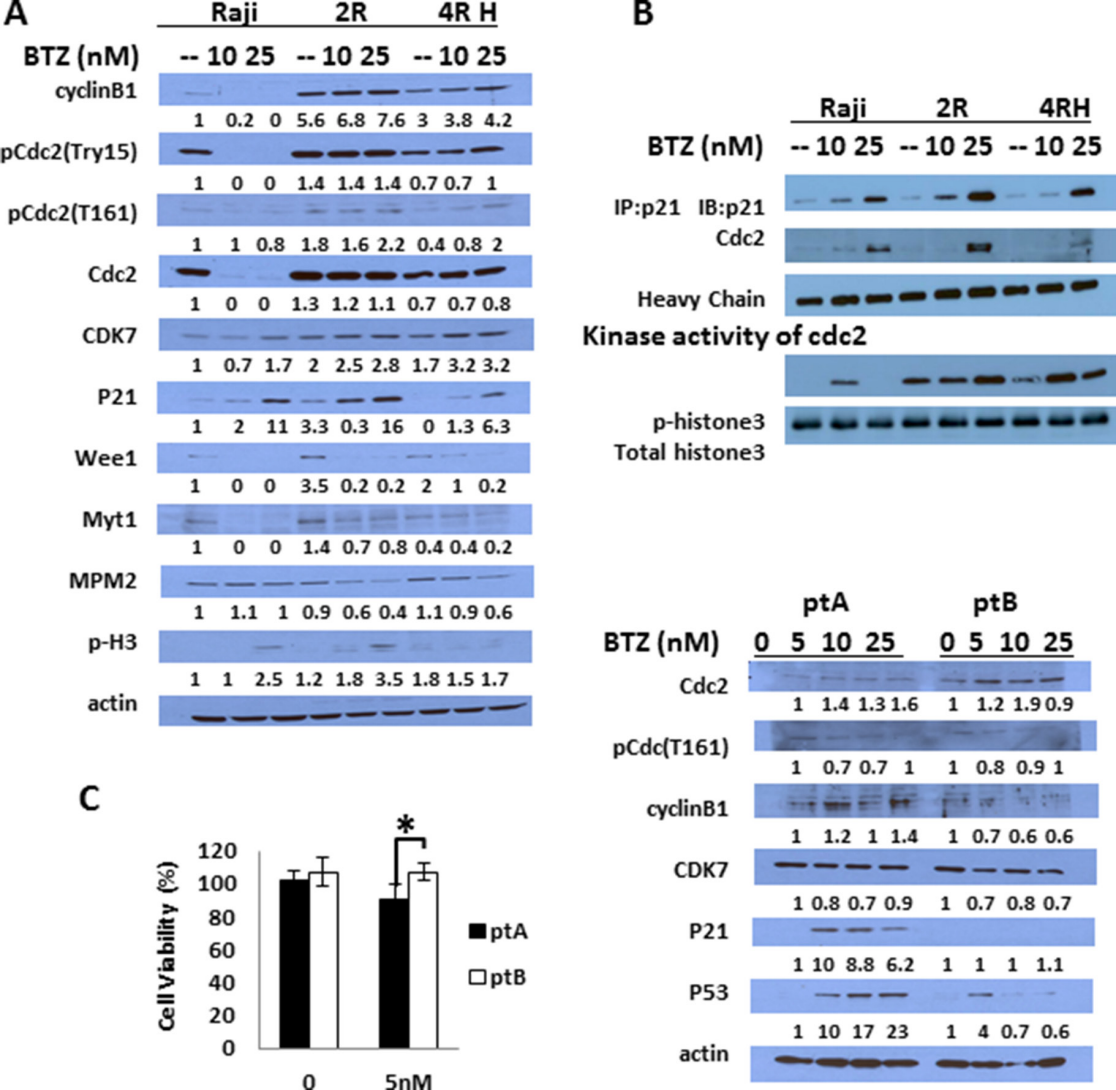
BTZ causes different effects on cell cycle regulated proteins and apoptosis proteins in RSCL and RRCL (**A**) cells were treated with BTZ 10 nM or 25 nM for 24 hrs, and cell exacts were prepared and analyzed by Western blot with the indicated antibodies to show changes in G2/M and G1/S cell cycle regulated proteins. β-actin was used as a loading control. (**B**) p21 was pulled down by anti-p21 antibody using co-immunoprecipitation protocol according to the Methods description with/without BTZ treatment, and CDC2 which was binding with p21 was detected by anti-CDC2 antibody. Heavy chain exposure was used as a loading control. The pulled down p21/CDC2 complexes were also used for an in vitro kinase assay by adding recombinant unphosphorylated histone 3 as its phosphorylation substrate. Phospho-histone 3 antibody was used to show the kinase activity of p21 pull down complex and total histone 3 antibody was used as control. (**C**) Similar changes in cell cycle regulatory proteins was observed in primary tumor cells isolated from a lymphoma patient sensitive to BTZ (pt A) ex vivo but not in a tumor cells isolated from a patient resistant to BTZ (pt B) (*represents P < 0.01). Each bar represents of mean ± SD of three independent of experiments. Patient A had de novo marginal zone lymphoma and patient B had relapsed follicular lymphoma.

### BTZ decreases cell senescence, induces G2/M-Phase arrest and mitotic catastrophe in RRCL, but not in RSCLs

*In vitro* exposure of RRCL and RSCL to BTZ decreased the number of cells in senescence (*P* < 0.01, Figure 2A). In BTZ-exposed RSCL, senescence cells were decreased by 75% (from 1 to 0.24 fold), whereas in BTZ-exposed RRCL, β-galactosidase activity decreased by 50.8% (1.2 to 0.61 fold, Raji 2R cells) and 40.4% (2.4 to 1.4 fold, Raji 4RH) in RRCL.

Following *in vitro* exposure to BTZ additional differences in the cell cycle distribution were observed between RSCL and RRCL (Figure 2B). In RSCL, we observed an increase in the percentage of cells at G1 phase (DMSO = 65.5 ± 3.8% versus BTZ = 80.6 ± 2.5%; *P* < 0.05) and a reduction at S-phase (DMSO = 26.0 ± 2.5% versus BTZ = 7.8 ± 1.4%; *P* < 0.05). However, there was no significant change in the number of cells distributed in the G2/M phase. A significant number of cells were found to be in the Sub-G1 region corresponding to apoptotic cells (DMSO = 6% versus BTZ = 12.7%; *P* < 0.01, data not shown). In contrast, BTZ exposure in RRCL, resulted in a reduction of cells in S-phase (Raji 2R DMSO = 41.4 ± 7.4% versus BTZ = 17.4 ± 5.6%, *P* < 0.05; Raji 4RH DMSO = 48.5 ± 3.1% versus BTZ = 13.9 ± 2.8%, *P* < 0.05; respectively) and an accumulation of cells in G2/M phase (Raji2R DMSO = 7.52 ± 4.8% versus BTZ = 35.78 ± 4.8%, *P* < 0.05; Raji 4RH DMSO = 4.6 ± 4.0% versus BTZ = 21.5 ± 5.5%, *P* < 0.05, respectively). An increase in cells at G1 was observed in Raji 4RH exposed to BTZ (DMSO = 46.7 ± 2.5% versus BTZ = 64.5 ± 3.1%; *P* < 0.05), but not in Raji 2R cells. In contrast to RSCL, no significant apoptosis (cells in sub-G1 region) was observed in RRCL at 24 hrs.

To correlate changes in cell cycle distribution observed in BTZ-exposed RRCL and cell division, we quantified the mitotic index in BTZ exposed RSCL or RRCL (Figure 2C). We demonstrated that BTZ induced mitosis in a dose- and time-dependent manner in RRCL and to a lesser degree RSCL. BTZ exposure increased the mitotic index in Raji 2R. We further confirmed changes in the mitotic index by immunofluorescence using a FITC-labeled anti-MPM2 antibody (Figure 2D). BTZ increased MPM2 positive cells in RRCL in a dose-dependent manner, but not in RSCL.

We demonstrated that, while caspase-dependent death pathways are executed in RSCL, caspase-independent death pathways play a role in BTZ-associated effects in RRCL [8]. Autophagy and mitotic catastrophe constitute alternative pathways of cell death upon stressful stimuli. In contrast to BH3-mimetics, BTZ does not induce autophagy [8, 32]. This led us to study if BTZ induced mitotic catastrophe in RRCL. Mitotic catastrophe is a form of cell death associated with uneven nuclear segregation during the mitosis phase. This distinct feature results from the formation of multi-nucleated cells and triggers cell-death [33]. Mitotic catastrophe can be visualized by con-focal microscopy following the double staining of α-tubulin (green) and nuclear structures (blue) (Figure 2E). We found that RSCL (Raji cells) underwent normal mitosis with/without BTZ treatment, but RRCL (Raji 2R cells) showed uneven nuclear segregation during mitosis and had multi-nucleate cells after BTZ (25 nM) exposure for 24 hrs. Similar changes were observed in Raji 4RH cells (data not shown).

### *In vitro/ex vivo* BTZ exposure induces the accumulation of G2/M regulatory proteins

BTZ exposure led to a dose-dependent accumulation of p21 in RRCL and to a lesser degree RSCL (Figure 3A). In addition, we found that UPS inhibition resulted in an up-regulation of CDK7 and a downregulation of CDC2 and cyclin B in RSCL but not in RRCL (Figure 3A). No significant changes in Weel and Myt1 levels were observed. To evaluate if p21 accumulation following BTZ exposure was functionally relevant, we performed co-immunoprecipitation studies and *in vitro* kinase activity assays. BTZ exposure increased p21-CDC2 protein-protein interaction (Figure 3B) in RRCL (and to a lesser degree in RSCL). Since CDC2 is a kinase that phosphorylates down-stream proteins (i.e. cyclin B, histone3, etc.) to initiate the mitotic phase, we studied the effects of BTZ on the kinase activity of p21/CDC2 complex using histone3 as a protein substrate. BTZ exposure in RRCL and RSCL increased the phosphorylation of histone3. Together our data indicates that BTZ upregulates p21, facilitates p21/CDC2 interaction, and increase its kinase activity leading cells to initiate the mitotic phase.

We then evaluated changes in key-regulatory proteins of the cell cycle in tumor cells isolated from two patients with B-cell lymphoma patients. *In vitro* sensitivity assays demonstrated that one patient with DeNove MZL (Pt. A) was sensitive to BTZ and the second patient with relapsed FL (Pt. B) was resistant (*P* < 0.001, Figure 3C). *Ex vivo* BTZ exposure in Pt. A and Pt. B primary tumor cells resulted in different changes in cell cycle regulatory proteins. Pt. A (BTZ-sensitive) exhibited a marked induction of p21 following BTZ drug exposure in contrast to Pt. B (BTZ-resistant). In addition, a marked up-regulation of p53 was also observed in Pt. A following BTZ therapy. No changes were observed in CDC2 levels following BTZ between Pt. A and Pt. B.

### Accumulation of p21 is required for BTZ anti-tumor activity in RRCL but not in RSCL

**Figure 4:**
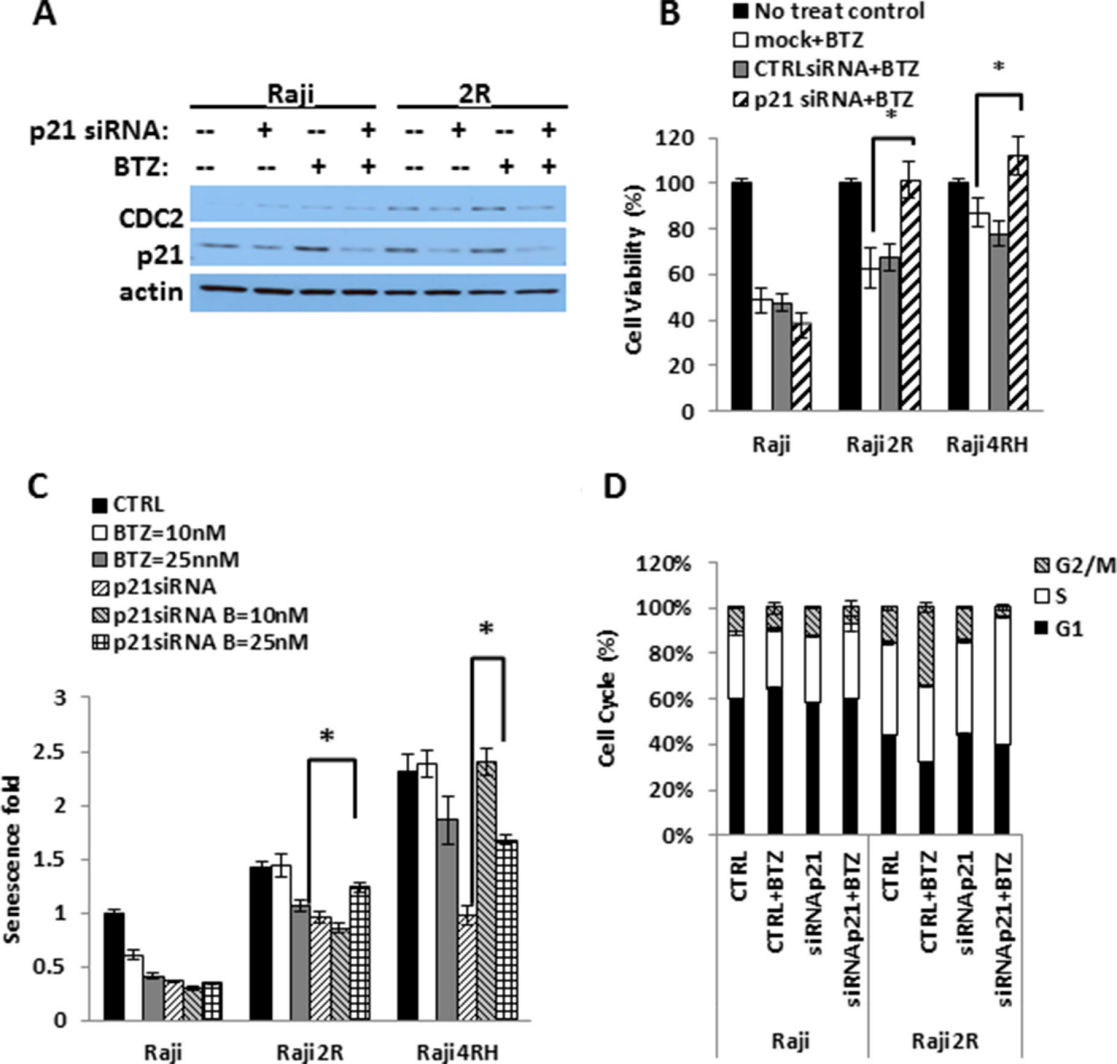
BTZ killing is p21-dependent in RRCL Endogenous p21 was knocked down using OnTARGET plus SMART pool siRNA against p21 and cells were exposed to different doses of BTZ. (**A**) cell lystates were harvest to shown endogenous p21 expression after knocking down by Western Blot. (**B**) cell viability assay was performed compared BTZ 25 nM 48 hrs killing effects in no siRNA control, non-specific siRNA control and p21 siRNA knockdown cells in RSCL and RRCL. (*represent P < 0.01). (**C**) Cell Senescence fold data was acquired according to the “Material and Methods” to compare the senescence change in the presence of BTZ with or without p21 expression. (**D**) cell cycle distribution of RSCL and RRCL p21siRNA knock down exposed to BTZ 25 nM 24 hrs. (*represents P < 0.01). Each bar represents of mean ± SD of three independent of experiments.

To further define the role of p21 accumulation and the induction of senescence and/or mitotic catastrophe following BTZ therapy, we silenced *CDKN1A* (Figure 4A). Transient siRNA knock down of p21 rescued RRCL but not RSCL to the cytotoxic effects of BTZ (Figure 4B). In addition, p21 knockdown abrogated the negative effects of BTZ in senescence of RRCL but not in RSCL (Figure 4C). Furthermore, we evaluated the impact of p21 down regulation in the cell-cycle distribution of RRCL and RSCL exposed to BTZ. As expected, siRNA knock down of p21 decreased the accumulation of BTZ-treated RRCL but not RSCL in G2M-phase (33.9 vs. 4.3%). At the same time, a compensatory accumulation of cells in the S-phase (33.4% vs. 56%) was observed in p21 knock out BTZ exposed RRCL (Figure 4D). Our data indicates that p21 up-regulation is a critical mediator to the senescence, cell cycle regulator and killing effects by BTZ in RRCL.

### BTZ potentiates the cytotoxic effects of M-phase cell cycle specific chemotherapeutic agents to RRCL and in primary tumor cells

**Figure 5:**
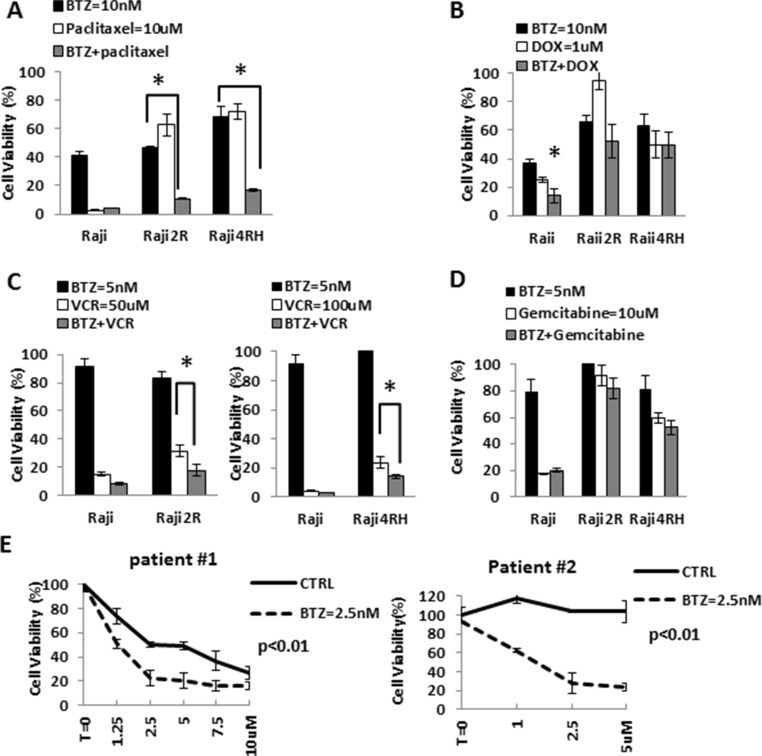
BTZ enhanced chemotherapy-induced killing effects in RRCL and cells derived from patients, but not in RSCL (**A**) Raji, Raji2R and Raji4RH were exposed to bortezomib (10 nM) for 24 hrs. and subsequently exposed to paclitaxel for 48 hrs. Cell viability was measured and the percentage of cell viability was calculated. Each point represents the mean of +/– SD of three independent experiments. A statistically significant (P < 0.01) decrease in cell viability in 2R and 4RH cells treated with bortezomib were seen compared to Raji treated groups. (**B**) Raji family cells pretreated with bortezomib 10 nM for 24 hrs, then incubated with doxorubicin for another 48 hrs. (**C**) Pretreated Raji family cells with bortezomib 5 nM for 24 hrs, and then exposed to vincristine 50 uM (Raji and Raji2R) to vincristine 100 uM (Raji4RH). Cell viability was addressed after 48 hrs. (**D**) Pretreated Raji family cells with bortezomib 5 nM for 24 hrs, and then cells were exposed to gemcitabine (10 uM) for 48 hrs. (**E**) Cells derived from patients ex vivo exposed to bortezomib 2.5 nM for 24 hrs. Pretreated cells were then incubated to paclitaxel at 2.5 uM, 5 uM, 10 uM for 48 hrs.

**Figure 6:**
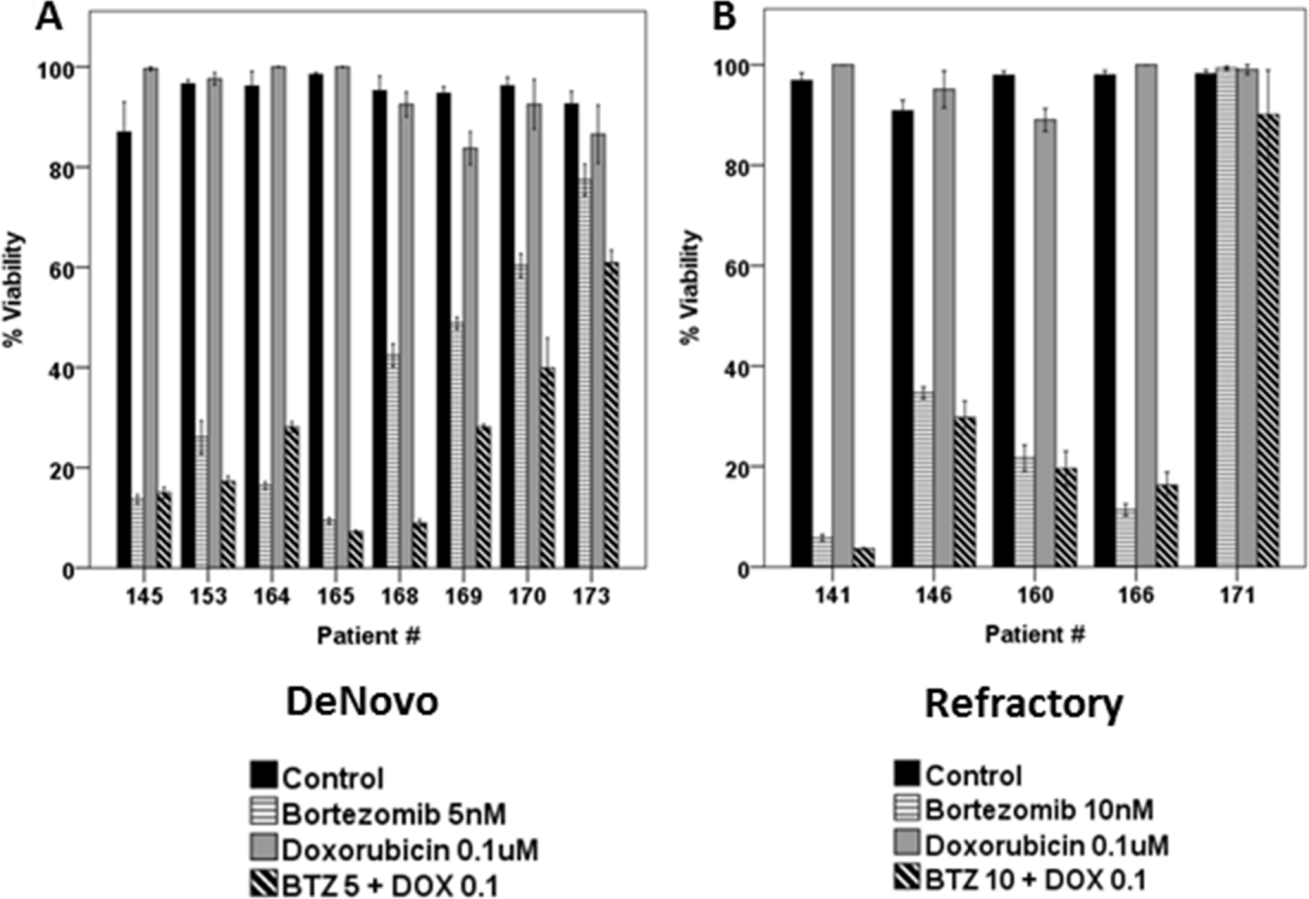
Combined BTZ and DOX treatment was effective in inhibiting cell viability in primary tumor cells isolated from patients (N = 13) Lymphoma cells derived from De novo patients (**A**) or refractory B-cell neoplasm (**B**) exposed to BTZ (5 or 10 nM) and doxorubicin (0.1 uM) for 48 h. Surrogate of viability was evaluated by the same method as mentioned previously.

Dunleavy et al. demonstrated that BTZ modulated the anti-tumor activity of systemic chemo-immunotherapy in patients with relapsed/refractory DLBCL [13]. Based on the effects of BTZ on the cell cycle of RRCL, we evaluated the therapeutic value of proteasome inhibition in the anti-tumor activity of M-cell cycle specific chemotherapy agents in RRCL. Pre-incubation of RRCL with BTZ re-sensitized not only RRCL but also primary tumor cells to cytotoxic effects of paclitaxel, doxorubicin and vincristine (Figure 5A, and 5D). No enhanced activity was observed when BTZ pre-treated RRCL were exposed to non-cell cycle specific agents (i.e. gemcitabine) (Figure 5D). Similar effects were observed in tumor cells isolated from B-cell lymphoma patients (Figure 5E). To further evaluate BTZ effects on the cytotoxic effects of chemotherapy drugs, we tested the anti-cancer activity of BTZ and doxorubicin combination in 13 primary tumor cell samples isolated from B-cell lymphoma patients (Figure 6A and 6B). A decrease of cell viability was observed in 75% (6/8) *de novo* samples and 40% (2/5) refractory samples exposed to BTZ and doxorubicin combined therapy. Descriptive statistics of lymphoma patients of Roswell Park Cancer Institute were included in Supplementary Material as Supplementary Table 1.

## DISCUSSION

Understanding the cellular events occurring following UPS inhibition will help to optimize the use of BTZ and likely other proteasome inhibitors. Rituximab resistance is associated with increased UPS activity, UPS-dependent Bax down-regulation, deregulation of apoptosis, and chemotherapy resistance [7]. We demonstrated that BTZ treatment of RRCL increased Bak levels partially restoring sensitivity to chemotherapy agents [7, 34]. Here, we characterize BTZ caspase-independent mechanisms of cell death.

Senescence is defined as a stage of cell proliferative arrest as a result of stressful environmental conditions such as DNA damage following exposure to anti-cancer drugs. Senescence was generally believed as a cell cycle arrest in G0/G1 stage. Recently, investigators reported that senescence was also triggered by p21 mediated G2/M phase cell cycle arrest [35–40]. Although during senescence cancer cells stop dividing, they maintain metabolic and synthetic activity and increased lysosome activity [41–43]. Most importantly, recent studies suggest that, by entering into cellular senescence, cancer cells evade the toxic effects of anti-cancer treatments. Influencing cellular senescence may be used to overcome therapy resistance in lymphoma patients [44–46]. Our data suggest that the repeated exposure of lymphoma cells to rituximab resulted in morphological and functional features suggesting an increase of senescence. *In vitro* exposure to BTZ decreased in a dose-dependent manner the number of cells in senescence. Our findings were more evident in Raji-2R cells than Raji-4RH cells, and this could be related to differences in the selective pressure method used to generate the rituximab resistant cell lines (rituximab alone vs. rituximab + human serum). More importantly, its effects on cell senescence appear to be p21-dependent. To our knowledge, this is the first report demonstrating that BTZ affects senescence in B-cell lymphoma cells. In human xenografts, the senescence phenotype was generated after retinoid or doxorubicin treatment [47, 48]. The presence of senescence cells in the tumor is a significant prognostic factor for the disease outcome. Induction of senescence after chemotherapy, radiation or hormone ablation correlates with poor outcomes [42, 49].

We demonstrated that BTZ induces G2/M cell cycle arrest and mitotic catastrophe in RRCL but not in RSCL. Similar effects had been observed in other pre-clinical models (i.e. non-small cell lung cancer, prostate cancer, Ewing's sarcoma, hepatocellular carcinoma cells, natural-killer (NK) cell lymphoma, vascular endothelial cancer, and other NHL cell lines) [15, 26–31]. Shen et al. demonstrated that in Bcl-2 deregulated natural killer (NK)-cell lymphoma cell lines, BTZ induced cell death by mitotic catastrophe as induction of apoptosis was significantly impaired in those cell lines due to extensive delayed turnover of Bcl-2 family members [29]. Similarly, in our RRCL (known to be deficient of Bak/Bax and therefore more resistant to apoptotic stimuli), BTZ induced both G2/M cell cycle arrest and mitotic catastrophe. On the other hand, RSCL underwent apoptosis following BTZ exposure, further stressing the existence of different mechanisms of cell death responsible for BTZ-associated biological activity.

P21 is a well-known CDK inhibitor of the G1-S-phase and is upregulated upon stress conditions [50]. Other functions of p21 include a role in cellular differentiation, senescence, and inhibition of apoptosis [26, 51, 52]. In RRCL, BTZ exposure resulted in an increase in the accumulation of p21, CDC2 and cyclinB in RRCL. Moreover, protein-protein interactions studies revealed and increase in the physical interaction between CDC2/p21 in RRCL after BTZ *in vitro* exposure when compared to the BTZ-exposed RSCL. CyclinB1 and CDC2 form a complex during G2 phase and its degradation by the UPS regulates the transition of cells into the metaphase [53, 54]. In our RRCL, BTZ exhibited similar effects: increase in CDC2/p21 interaction, downstream phosphorylation of substrate proteins (i.e. histone3), and an increase in mitotic index. We hypothesized that BTZ may overcome acquired resistance to chemotherapy drugs by a p21 dependent-mechanism in RRCL. Dash et al. reported that in G2/M transition, hyper-phosporylated p21 forms a complex with CDC2 and therefore promoting CDC2-kinase activity [52]. Transient silencing of p21 decreased CDC2 expression and rescued RRCL but not RSCL to the cytotoxic effects of BTZ. Moreover, cell cycle distribution was different in BTZ exposed p21-intact versus p21-knockout RRCL. An increase in S phase and decrease in G2M phase was observed in p21 knockout RRCL exposed to BTZ. The increase in S phase following BTZ exposure in p21 deficient RRCL could be the result of protein interaction between cell cycle regulatory proteins negatively regulated by p21 (i.e. CDCK2 or cyclin E). We found a correlation between the activity of BTZ and p21 stabilization in tumor cells isolated from B-cell lymphoma patients. While we only studied 2 samples (one BTZ-sensitive and one BTZ-resistant sample), our findings suggest that p21 stabilization plays a significant role in the anti-tumor activity of BTZ in B-cell lymphoma. This finding will require further validation in a larger sample of primary tumor cells isolated from B-cell lymphoma patients.

Mitotic catastrophe is a form of cell death with aberrant mitosis occurring during G2/M phase, characterized by the asymmetrical segregation of chromatid clusters and/or the formation of large cells with multiple micronuclei. It is tightly controlled process by several key regulatory proteins including CDC2, cyclin B, aurora kinase, p53 and members of BCL-2 family proteins. Upon stressful conditions cells deficient of Bcl-2 family members fail to undergo programed cell death. On the other hand, such cells can die via the execution of mitotic catastrophe [29, 33, 55].

Our work defines additional mechanisms of cell death triggered by BTZ, especially in lymphoma cells resistant to apoptotic stimuli. Our work suggests that BTZ possesses various mechanisms-of-action and that their execution varies according to the apoptotic threshold of the cell and on the balance of Bcl-2 family members. In lymphoma cells with “intact” apoptotic machinery, induction of apoptosis is the primary mechanism-of-action of BTZ (i.e. RSCL). On the other hand, in lymphoma cells with deregulated apoptotic potential (i.e. RRCL) induction of mitotic catastrophe and cell cycle arrest may play more dominant roles in BTZ-associated anti-tumor activity. While autophagy had been suggested as an alternative caspase-independent mechanism of cell death for some anti-cancer agents, we could not demonstrate autophagy in BTZ exposed RRCL [8].

Different therapeutic approaches may optimize BTZ activity clinically. In one, BTZ could be used to modulate Bcl-2 family members levels and increase the number of cells undergoing apoptosis following standard chemotherapy agents in B-cell lymphoma. Alternatively, in refractory B-cell lymphoma with impaired apoptotic machinery, the combination of BTZ and cell-cycle specific chemotherapy agents or novel cell cycle inhibitors may improve outcomes for this subset of patients.

## MATERIALS AND METHODS

### Cell Lines and primary tumor cells

A panel of rituximab-sensitive cell lines (RSCL) or RRCL was used. RRCL were created from each RSCL by repeat exposure of cells to escalating doses of rituximab in the presence (4RH) or absence (2R) of human complement. Individual clones were isolated and expanded. These RRCL have been previously described and characterized [6, 8]. We previously demonstrated that both types of RRCL are known to down-regulate surface CD20, up-regulate complement inhibitory proteins (CD55 and CD59), up-regulate the components of the UPS and deregulate Bcl-2 family member proteins leading to an increase in the resistance to apoptotic stimuli [6, 8].

Neoplastic B-cells were isolated from pre-treatment biopsy tissue obtained from patients with B-cell lymphoma [diffuse large B-cell lymphoma (DLBCL), follicular lymphoma (FL), mantel cell lymphoma (MCL), marginal zone lymphoma (MZL) and chronic lymphocytic leukemia (CLL)] receiving therapy at Roswell Park Cancer Institute (RPCI) as previously described [32]. Samples from patient biopsy specimens were procured under Institutional Review Board (IRB) approved RPCI protocols. RSCL, RRCL or primary tumor cells were plated all a cell density of 0.5 × 10^6^ cells/ml unless otherwise specified.

### Antibodies and reagents

Doxorubicin was purchased from Bedford Laboratories (Bedford, OH); BTZ, paclitaxel, vincristine, and gemcitabine were provided by RPCI Pharmacy. Primary mouse anti-human antibodies raised against p21, cyclin-B, CDC2, Weel, Myt1, phospho-H3, CDK7, cyclin-A, CDK2, CDK4, CDK6, and actin were obtained from Cell Signaling (Boston, MA). The fluorescein isothiocyanate (FITC)-labeled anti-phosphor-Ser/Thr-Pro (MPM2) antibody was obtained from Millipore (Billerica, MA); the anti-PARP-1 antibody was purchased from BD Pharmigen (San Jose, CA), and the anti-tubulin-Alexa 488-conjugated monoclonal antibody from Invitrogen (Grand Island, NY). Propidium iodide (PI), RNase, Triton X-100, trypan blue, histopaque, and Wright Giemsa stain were obtained from Sigma-Aldrich Inc. (St. Louis, MO), and Q-VD-OPh from MBL International (Woburn, MA). The Cell Titer-Glo Luminescent Viability Assay was purchased from Promega (Madison, WI).

### *In vitro* effects of BTZ on the viability of NHL cell lines

RRCL or RSCL were exposed to escalating doses of BTZ (10–25 nM) or vehicle control (DMSO) for 24 or 48 hrs. with or without Q-VD-OPh (5 μM). At each time period cell viability was determined by measuring ATP content changes using the Cell Titer-Glo Luminescent Viability Assay. Percentage of viability was defined as the absorbance reading relative to DMSO exposed cells.

### Induction of senescence following *in vitro* exposure of RRCL to BTZ

In order to determine if senescence contributed to the anti-tumor activity of BTZ, RSCL and RRCL were exposed overnight to BTZ (25 nM) or DMSO. Senescence-associated β-galactosidase staining was performed with the Senescent Cells Staining Kit (Sigma, St. Louis, MO) according to the manufacturer's instructions. Photographs were taken with Nikon microscope. The quantitative senescence assay was performed using the 96-well Cellular Senescence Assay Kit (Cell Biolab Inc., San Diego, CA). Fluorescence measurement was performed on Opsys MR micro-plate reader (Thermo system, Chantilly, VA) with a 355 nm/460 nm filter set.

### *In vitro* effects of BTZ in the cell cycle, mitotic index, and induction of apoptosis in B-cell lymphoma

RSCL, RRCL and primary tumor cells were exposed *in vitro* to BTZ (10 nM) for 24 hrs. Subsequently, cells were harvested, washed with phosphate buffered saline (PBS), and fixed with ice cold 70% ethanol at –20°C for 30 minutes. Then, cells were incubated with 5 μg/ml RNase for 30 minutes at room temperature and stained with PI (5 μg/ml) for 1 h. Differences in cell cycle or induction of apoptosis (cells in Sub-G1 region) were determined by flow cytometry.

To study the differences in mitotic index between RSCL and RRCL, the cells were exposed to BTZ or DMSO for 24, 48, and 72 hrs. Samples were collected and stained with 50% Wright-Giemsa solution. The mitotic index was calculated using the following formula: Mitotic index=mitotic cells (staining cells)/total cell number*100%.

To determine whether cells enter into mitosis, RSCL and RRCL were exposed to BTZ (10 or 25 nM) *in vitro* for 24 hrs. Cells were fixed with 1 ml 2% formaldehyde at room temperature (RT) for 10 minutes. Cells were re-suspended in PBS+0.1% triton x100 (2 ml) for 30 minutes and then incubated for 1 hr with the FITC-labeled MPM2-2 antibody. Flow cytometry analysis was performed to test the different MPM2 expression levels in the cells (fold change normalized to RSCL untreated cells).

### Changes in the expression of several Bcl-2 family members or key regulatory proteins of the cell cycle in RSCL or RRCL after exposure to BTZ

RSCL, RRCL or primary tumor cells were exposed *in vitro* to BTZ (10 or 25 nM) or control for 24 hrs. Changes in cell cycle regulatory proteins (i.e. p21, cyclin-B, CDC2, Weel, etc.) or Bcl-2 family members expression (Bak, Bax, Mcl-1, Bcl-2, etc.) were determined by Western blotting.

### Changes in tubulin localization induced by BTZ in B-cell lymphoma cells

RSCL and RRCL were exposed to BTZ at 25 nM for 24 hrs, fixed with 1.5% formaldehyde for 10 minutes, and cell membranes were permeabilized with ice-cold MeOH at RT for 10 minutes. Cells were washed twice in PBS containing 1% bovine serum albumin (BSA) and tubulin staining was performed using a FITC labeled mouse anti-human tubulin monoclonal antibody (dilution 1:100) overnight in the dark. Finally stained cells were washed three times in 1%-BSA-PBS, re-suspended in mounting media and affixed into Alcian blue cover slips. 4′,6-diamidino-2-phenylindole (DAPI) (Vector Laboratories, Burlingame, CA) was used for nuclear staining. Photographs were taken using a Leica TCS SP2 Spectral confocal fluorescence imaging system.

### Effects of BTZ on the interaction between p21 and CDC2 in RRCL

After BTZ exposure (10 nM, 25 nM × 24 hrs), 5 × 10^6^ RSCL or RRCL were harvested, washed with PBS, lysed with 0.5 ml ice-cold 1X cell lysis buffer containing AEBSF Hydrochloride (500 μM), aprotinin (150 nM), E-64 protease inhibitor (1 μM), EDTA (0.5 mM) leupeptin (1 μM), sodium pyrophosphate decahydrate (50 nM), sodium orthovanadate (50 nM) (Proteasome inhibitors cocktail set 1 and phosphatase inhibitor cocktail set V (Millipore, Billerica, MA) and incubated for 5 minutes. Cell lysates were clarified by ultracentrifugation at 15,000-× g for 20 minutes. The supernatants were collected for each sample and incubated with anti-p21 antibody or control at 4°C for 1.5 h with gentle rotation; twenty μl PBS-washed protein A agarose beads (Cell Signaling, Boston, MA) were added into each sample and incubated for another 2 hrs. at 4°C. The beads were washed 3 times with 1 ml of lysis buffer. Antibody-bound complexes were eluted by boiling in 2× Laemmli sample buffer and resolved by 12% SDS-polyacrylamide gel electrophoresis and transferred onto PDVF membranes. Membranes were blocked in TPBS including 2% non-fat milk 4°C overnight, probed with a mouse anti-human CDC2 antibody fallowed by proper secondary antibody. The immunoreactive proteins were visualized by enhanced chemiluminescence (SuperSignal; Pierce).

In addition, we performed an *in vitro* kinase assay. Antibody-bound (p21-IP samples) samples were washed twice with 500 μl 1X Kinase Buffer (Cell Signaling, Boston, MA) and re-suspended in 1X Kinase buffer supplemented (50 μl) with 200 μM ATP (Cell Signaling, Boston, MA) and kinase substrate histone3 (H3) recombinant protein (1 μl)(Cell Signaling, Boston, MA). The samples were well mixed and incubated at 30°C for 30 minutes. To terminate reaction, 50 μl of 2X SDS sample buffer were added. Samples were vortexed, micro-centrifuged for 30 seconds and heated at 100°C for 5 minutes. *In vitro* kinase activity was determined by analyzing the phosphorylation status of H3 using a phospho-specific antibody against H3 (Ser10) by Western Blot.

### The role of p21 on BTZ anti-tumor activity in RRCL

Knockdown of p21 was achieved using ON-TARGET plus SMART pool siRNA containing a mixture of 4 siRNAs designed to specifically target *CDKN1A* (Dharmacon, Lafayette, CO). Non-targeting SMART pool siRNA (Dharmacon) was used as a negative control. Efficient knockdown of p-21 was confirmed by Western blotting. Once conditions were optimized, RRCL were transfected with 2 μg of siRNA targeting *CDKN1A* or control using an Amaxa Nucleofector (Lonza laboratories, Anaheim CA) and cells were incubated with BTZ (0– 25 nM) for an additional 24 or 48 hrs. Differences in cell viability, senescence and mitotic index were determined by ATP quantification, β-galactosidase and Wright-Giemsa staining respectively.

## SUPPLEMENTARY MATERIALS FIGURES AND TABLES


