# Supplementary material for: Mitotic catastrophe and cell cycle arrest are alternative cell death pathways executed by bortezomib in rituximab resistant B-cell lymphoma cells
Source: Oncotarget. 2016 Dec 31;8(8):12741–53. doi: 10.18632/oncotarget.14405 (PMC5355050; doi:10.18632/oncotarget.14405)
Supplement: Supplementary file 1 [file oncotarget-08-12741-s001.pdf]

Mitotic catastrophe and cell cycle arrest are alternative cell death pathways executed by bortezomib in rituximab resistant B-cell lymphoma cells

Supplementary Materials

Supplementary Table 1: Descriptive statistics of the patients from whom the primary tumor cells were isolated

	All patients	De novo	Refractory
Number (%)	13	8 (64.2%)	5 (35.7%)
Mean age	59	64.3	50.8
Sex Subtype	3/10 (23%/77%)	3/5 (37.5%/62.5%)	0/5 (0%/100%)
CLL F/M	5 (38.4%)	3 (37.5%)	2 (40%)
MZL	7 (53.8%)	5 (62.5%)	2 (40%)
DLBCL	1 (12.5%)	0	1 (20%)
